# Recent Strategies to Develop Conjugated Polymers for Detection and Therapeutics

**DOI:** 10.3390/polym15173570

**Published:** 2023-08-28

**Authors:** Yutong Li, Ruilian Qi, Xiaoyu Wang, Huanxiang Yuan

**Affiliations:** 1 Department of Chemistry, College of Chemistry and Materials Engineering, Beijing Technology and Business University, Beijing 100048, China; 2 School of Materials Science and Engineering, University of Science and Technology Beijing, Beijing 100083, China

**Keywords:** conjugated polymers, pathogenic infections, cancer, detection, therapy

## Abstract

The infectious diseases resulting from pathogenic microbes are highly contagious and the source of infection is difficult to control, which seriously endangers life and public health safety. Although the emergence of antibiotics has a good therapeutic effect in the early stage, the massive abuse of antibiotics has brought about the evolution of pathogens with drug resistance, which has gradually weakened the lethality and availability of antibiotics. Cancer is a more serious disease than pathogenic bacteria infection, which also threatens human life and health. Traditional treatment methods have limitations such as easy recurrence, poor prognosis, many side effects, and high toxicity. These two issues have led to the exploration and development of novel therapeutic agents (such as conjugated polymers) and therapeutic strategies (such as phototherapy) to avoid the increase of drug resistance and toxic side effects. As a class of organic polymer biological functional materials with excellent photoelectric properties, Conjugated polymers (CPs) have been extensively investigated in biomedical fields, such as the detection and treatment of pathogens and tumors due to their advantages of easy modification and functionalization, good biocompatibility and low cost. A rare comprehensive overview of CPs-based detection and treatment applications has been reported. This paper reviews the design strategies and research status of CPs used in biomedicine in recent years, introduces and discusses the latest progress of their application in the detection and treatment of pathogenic microorganisms and tumors according to different detection or treatment methods, as well as the limitations and potential challenges in prospective exploration.

## 1. Introduction

Conjugated polymers (CPs) are a kind of carbon chain skeleton organic macromolecular compound composed of one or more conjugated units connected by covalent bond polymerization, which form a large π-bond. The large π-electron delocalization and the conjugated backbone [1] composed of multiple electron donor-acceptor (D-A) repeating units make CPs have strong light-trapping ability and excellent photophysical properties. Moreover, most CPs have the advantages of low cost [2,3], easy modification and functionalization [4], biological safety [5], and biodegradability [6]. They have been widely used in research from photoconducting devices to biological fields.

As is known to all, pathogenic microbes have always constituted a huge threat to global public health [7]. Pathogenic infections may lead to serious diseases such as diarrhea, inflammation, and septicemia, which threaten life, [8] and may even cause a potentially large number of deaths each year [9]. The early use of antibiotics showed a good bactericidal effect and made a great contribution to solving pathogenic infections, but the abuse and improper use of antibiotics led to the emergence of drug-resistant strains [10] and even the multi-drug resistant super-strains. It is indicated that pathogenic infections, especially those that result from new drug-resistant bacteria, will become the second largest cause of death in the world [11]. In addition, the World Health Organization (WHO) has declared that pathogen resistance is one of the top 10 global public health threats that humans will be confronted with [12]. Thus, in the face of the above severe crisis, the efficient detection of pathogens and treatment of infections that do not easily cause drug resistance are particularly urgent and crucial.

However, there are still many limitations in the traditional detection methods of pathogenic bacteria and therapeutic materials instead of antibiotics. For example, the method based on microbial culture has a long culture time (48–72 h) and is susceptible to contamination [13]. Antibody detection requires a large number of antigens, and the accuracy of the results is unstable, prone to false positives, and takes a long time [14]. Multiple polymerase chain reaction (PCR)-based molecular diagnostics and mass spectrometry require a high level of expertise and equipment for testing [15]. On the other hand, antimicrobials that replace antibiotics can currently be divided into three categories: natural antimicrobials, inorganic antimicrobials, and organic antimicrobials [16]. Natural antimicrobials such as chitosan, bacteriophage [17], catechol [18], sorbic acid, etc., have low bactericidal rates without specificity and cannot be used in a broad spectrum and long term. There is also a type of natural antibacterial peptide molecule, which can be easily removed from the body and cannot be transported to the target with accumulation, resulting in poor bactericidal effect and low utilization [19]. Inorganic antimicrobials are mostly materials based on metals such as Cu, Ag, and Zn, which are usually difficult to prepare and have poor biocompatibility. However, organic antimicrobials such as quaternary ammonium salts (QAS) [20] and phenols have problems such as easy hydrolysis, poor heat resistance, and a short validity period. In order to solve these problems, it is also essential to continue to explore antimicrobial materials with excellent antibacterial effects, good biocompatibility, and specificity for their application in practice. Therefore, it is necessary to develop more efficient, simple, cheap, and sensitive biosensing methods and new antibacterial agents with targeted and excellent bactericidal effects to detect and kill pathogens.

Cancer, another disease that is more serious than pathogenic infection, is also a global human public health problem [21] and one of the main causes of death worldwide. According to the estimate of the WHO, nearly 100,000 people will die from cancer in 2020 [22] seriously endangering human life and health. At present, conventional treatment and diagnosis methods for cancer include surgery [23], chemotherapy [24], radiotherapy [25], computed tomography (CT), magnetic resonance imaging (MRI), etc., which have many limitations such as high-risk, easy recurrence, poor prognosis, many side effects, high toxicity, and low resolution [26]. In addition, the continuous development of drug resistance seriously limits the therapeutic effect, and the number of cancer patients and deaths continues to rise globally [27]. Therefore, there is an urgent necessity for biological materials and therapeutic strategies that can overcome drug resistance, accurately diagnose, have excellent curative effects, and have a good prognosis.

**Figure 1:**
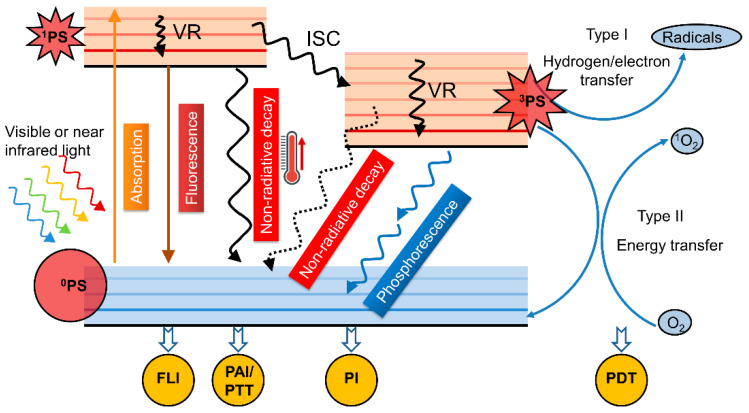
Illustration of a Jablonski diagram. (PS: photosensitization agents. VR: vibrational relaxation. FLI: fluorescence imaging. PAI: photoacoustic imaging. PTT: photothermal therapy. ISC: intersystem crossing. PI: phosphorescence imaging. PDT: photodynamic therapy).

With the progress of scientific research and technology, the emergence of advanced materials with excellent properties has brought hope to solve the above needs and challenges. Among them, CPs have many advantages mentioned above, and most reported CPs for biomedical applications have excellent biocompatibility, such as water-soluble conjugated polyelectrolytes with various backbones, including poly(fluorene-co-phenylene) (PFP), poly(phenylenevinylene) (PPV), poly(phenylene ethynylene) (PPE), polythiophene (PT), and polydiacetylene (PDA) [28] which have great potential as a class of new material to be widely used in biosensing, antibacterial and anticancer fields. such as biosensors, antibacterial and anticancer therapy [29,30]. CPs can utilize Förster resonance energy transfer (FRET), signal amplification, and excellent light conversion capabilities for many chemical and biomolecular sensing analyses, as well as cellular and animal-level imaging and therapy [31]. Therefore, CPs have been widely used as a probe or a light therapeutic agent based on the conversion of photon energy activated by light into optical signals or therapeutic effects. This process can be explained as shown in Figure 1 [32]. As photosensitization agents, CPs can absorb energy to transition from the ground state (S0) to the high-energy and short-lived excited singlet state (Sn) under the excitation of appropriate wavelength of light, then undergo a rapid internal conversion (IC) process to the lowest energy level of the singlet excited state (S1), and finally go through three different energy dissipation paths from the S1 state to S0 including (1) fluorescent emission of radiative transitions, which can be used for fluorescence imaging (FLI) to guide detection, diagnosis and drug administration [33], (2) intersystem crossing (ISC) to a excited triplet state, followed by transferring hydrogen/electron/energy to nearby oxygen/biomolecules to produce cytotoxic reactive oxygen species (ROS) comprised of radicals (type I way) and singlet oxygen (type II way), which can be used in photodynamic (PDT) therapy [34], and (3) heat or sound wave generation through non-radiative decay, which can be used for photothermal (PTT) therapy or photoacoustic imaging (PAI) [35]. CPs-based phototherapy overcomes antibiotic resistance and has the advantages of high spatial and temporal accuracy, noninvasiveness, low toxicity, and few side effects, thus showing unlimited biomedical potential in the diagnosis and treatment of pathogens and tumors [36].

Herein, the design strategies and research status of CPs in the biomedical field in the recent four years are reviewed, and the research progress of CPs in the detection and treatment of pathogenic microorganisms and cancer by different methods is summarized. (Table 1) It focuses on the application of CPs in pathogen detection by fluorescence and colorimetry, as well as the antibacterial and antitumor application of CPs by phototherapy and many different methods. In addition, the emerging strategy of integrated diagnosis and treatment is also introduced and discussed. In the end, the limitations and potential challenges of CPs in the future development of biomedicine are discussed and prospected.

## 2. Conjugated Polymers for Pathogenic Microorganism Detection

### 2.1. Fluorimetry

As fluorescence imaging technology based on fluorescent materials has made great progress in biological detection [66,67,68], the fluorescence detection method has become a routine research technology for pathogen detection due to its advantages of high resolution, short time consuming, excellent specificity, and high sensitivity. Because of the superior photophysical properties of CPs, they are often used as fluorescent probes for the detection of pathogenic microorganisms.

**Figure 2:**
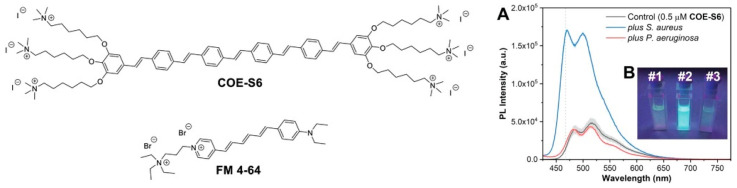
Chemical structures of fluorescent probes and the (**A**) spectra and (**B**) visual color detection results of *S. aureus* and *P. aeruginosa*. Reprinted with permission from ref. [37]. Copyright 2020 Wiley-VCH GmbH.

The Gram stain detection method based on common dyes has limitations such as complicated steps and poor fluorescence resulting in inaccurate results. Zhou et al. [37] realized the detection and typing of Gramella based on the fluorescence method by using a conjugated oligomer electrolyte (COE-S6) (Figure 2). It is formed by oligomerization of phenylenevinylene. Incidentally, the conjugated oligomer is a polymer composed of fewer conjugated repeating units, which are contained in a conjugated polymer. In the presence of COE-S6, the fluorescence enhancement of Gram-positive (G^+^) bacteria fluid can be visually detected in situ, while the lipopolysaccharide outer membrane of Gram-negative (G^−^) bacteria obstructed the embedding of COE-S6, leading to the almost negligible fluorescence intensity changes. In the mixed sample of G^−^ bacteria and G^+^ bacteria, the sensitivity and specificity of COE-S6 against G^+^ bacteria are much higher than that of commercially available probe FM-4-64. This work explored a simple and accurate method for gram bacteria typing based on a conjugated fluorescent probe with membrane specificity.

**Figure 3:**
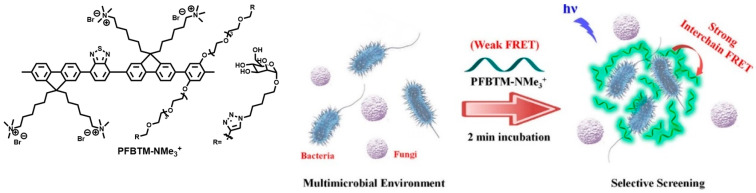
The structure of PFBTM-NMe_3_^+^ and the illustration of its selective detection of bacteria in mixed samples. Reprinted with permission from ref. [38]. Copyright 2020 American Chemical Society.

Most of the actual samples that need to be tested and distinguished contain a variety of microorganisms, so the detection of microorganisms in mixed samples is of great significance [69]. A lot of efforts are made to develop conjugated polymer-based fluorescence detection methods to accurately distinguish pathogen species in mixed samples. Hussain et al. [38] designed a water-soluble cationic CP (PFBTM-NMe_3_^+^) using polyfluorene derivative doped with benzothiazole (BT) groups as the main chain (Figure 3). The side chain was modified with the targeting group mannose and the QAS group which provided a positive charge and improved water solubility. The polymer uses a FRET mechanism to rapidly distinguish between bacteria and fungi based on differences in the membrane composition and surface charge of pathogenic microbes. When the polymer interacted with G^−^ bacteria *E. coli*, G^+^ bacteria *S. aureus*, and *C. albicans*, respectively, it showed a significant and rapid fluorescence response to *E. coli*, but a weak response to *S. aureus* and almost no response to *C. albicans*. The reason is that the surface of *E. coli* has lectin FimH that can bind to mannose and the most negative charges that could absorb positively charged PFBTM-NMe_3_^+^ to cause aggregation to exhibit strong FRET and fluorescence changes. However, *S. aureus* with less negative charges and *C. albicans* with the least negative charges as well as lack of lectins showed weak aggregation fluorescence and negligible fluorescence changes, respectively. In addition, in the detection of mixed samples of *E. coli* and *C. albicans*, PFBTM-NMe_3_^+^ showed certain aggregation after binding to *E. coli* and exhibited specific, rapid, and sensitive fluorescence responses. Therefore, through the design of different binding strategies between the synthetic polymer and various pathogens, it is possible to realize rapid and sensitive fluorescence imaging and identification of bacteria and fungi in mixed pathogen samples, giving new impetus to the research of early clinical diagnosis of bacterial infection.

**Figure 4:**
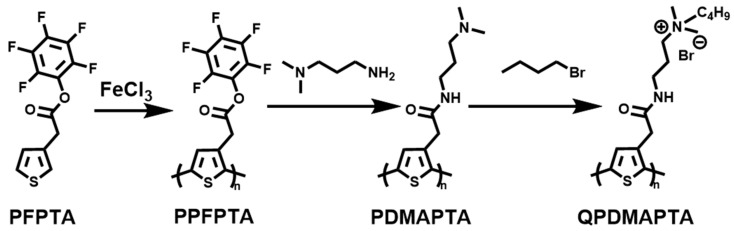
The synthetic route of QPDMAPTA. [39].

The conjugated polymer polythiophene derivatives with high conjugation and flexible structure have been explored for fluorescence imaging due to their excellent photophysical properties, but the low solubility limits their applications in diagnosis and therapeutics. Ren et al. [39] solved this problem by modifying QAS groups in the side chain of polythiophene. They polymerized a thiophene monomer (PFPTA) to obtain polymer PPFPTA, which was then coupled with a propylenediamine derivative followed by quaternizing with 1-bromobutane to obtain an active cationic polythiophene derivative (QPDMAPTA) (Figure 4). The conjugated polymer not only increases the solubility but also introduces a positive charge that can bind to the membrane of pathogenic bacteria through hydrophilic and electrostatic interactions. When the G^+^ bacteria (*S. aureus*, *S. epidermidis*, and MRSA) and G^−^ bacteria (*E. coli* and *P. aeruginosa*) were incubated with QPDMAPTA, green fluorescence was observed in the G^+^ bacteria groups, while the fluorescence of G^−^ bacteria was negligible. This comparison between the presence and absence of fluorescence can be used for the typing detection of Gramella. In addition, after centrifuging and washing the bacteria solution co-cultured with the polymer, it can be observed that the precipitation color changes to a darker yellow or brown in the G^+^ bacteria groups, which can be used to realize the rapid visual observation of the type of Gramella. Therefore, the synthetic QPDMAPTA has a bright prospect in the application of detection and differentiation of G^+^ bacteria, and this work has enabled the potential application of the fluorescence method and rapid visual bacteria typing diagnosis.

### 2.2. Colorimetry

Colorimetric sensors are widely used for detection due to their superiorities of low cost, high sensitivity and specificity, and visibleness (even to the naked eye) [70]. They are simple to use and do not require professional training and expensive instruments [71]. In recent years, the emergence of advanced conjugated polymer materials with excellent photophysical properties has greatly promoted the development of colorimetric sensors for the detection of pathogenic bacteria [72].

**Figure 5:**
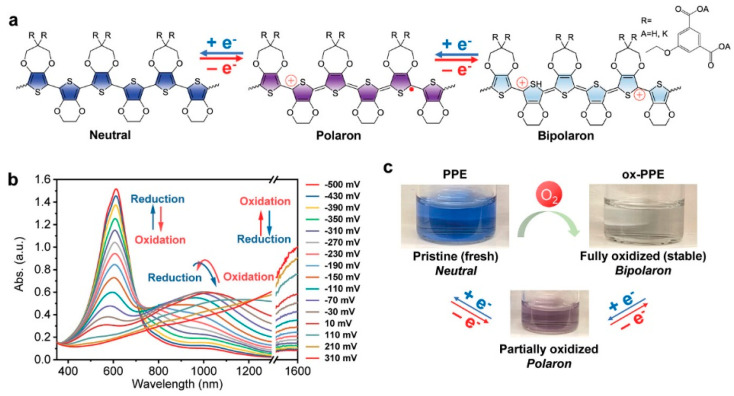
PPE in different oxidation states for detection. (**a**) The illustrated structures, (**b**) the spectra and (**c**) visual color detection of different oxidation states. Reprinted with permission from ref. [40]. Copyright 2020 Wiley-VCH GmbH.

Wu et al. [40] designed a polythiophene derivative (PPE), a water-soluble electrochromic copolymer, as a novel sensor for active bacterial metabolism. Due to the low oxidation potential of PPE, it can be doped by oxygen in the natural environment to generate an oxidation state (ox-PPE). ox-PPE could be de-doped by active bacterial metabolites such as cysteine and glutathione (Figure 5). The reduction process was accompanied by a visual color change of ox-PPE, that formed the blue uncharged conjugated polymer to the purple partially oxidized free radical cationic conjugated polymer (i.e., polaron) and then to the light blue oxidized bicationic conjugated polymer (i.e., bipolaron). The change was measured in a high-throughput manner with a 96-well microplate reader showing a significant change in the absorption spectra. The color change of ox-PPE was observed by controlled addition of cell-free supernatant, while no color change occurred in cell-membrane-bound ox-PPE, demonstrating that the main mechanism of reduction can be attributed to extracellular metabolites, especially with high sensitivity and specificity toward cysteine and glutathione. Additionally, ox-PPE is capable of distinguishing between G^−^ and G^+^ bacteria, as well as discriminating drug-resistant strains, and is used to assess sensitivity to various antibiotics. This simple, economical, high-throughput, and rapid approach opens up a new opportunity for personalized diagnosis and prescribing of infectious diseases.

**Figure 6:**
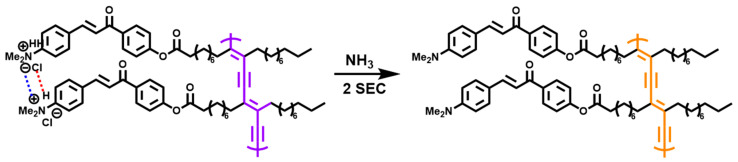
The reaction of PDA with metabolized ammonia gas for visual bacterial detection [41].

Manikandan et al. [41] synthesized chalcone head group modified polydiacetylene and realized bacterial visual detection by using color transformation of its stimulation response to bacterial metabolite ammonia after hydrochloric acid treatment (Figure 6). Specifically, polydiacetylene (PDA) is a type of CPs synthesized with 1, 3-butanediyne as a monomer that has a unique color and fluorescence transformation, usually appearing blue and changing to red when subjected to external stimuli such as pH, temperature, mechanical strain, and molecular interactions. Only when the yellow chalcone diacetylene film under the action of hydrochloric acid (HCl), by adjusting the electrostatic ion pair interaction between the adjacent chalcone head groups, hydrogen bonding, and intramolecular π-π stacking, effectively promote the appropriate arrangement of the diacetylene unit of the monomer (colorless), the diacetylene polymerization forms a conjugate PDA network structure (purple). However, when ammonia is encountered, HCl residues are removed, and the electrostatic interaction between chalcone heads and hydrogen bonds are destroyed, resulting in structural transformation and color changes. In addition, the polymerized membrane treated by HCl has reversible thermochromism in the temperature change of −50 to 20 °C (blue turning purple) and 20 to 50 °C (purple turning orange). First, it is attributed to the strong intramolecular interaction between HCl and chalcone groups, which promotes the flexibility of the structure and can produce different colors even at very low temperatures. At high temperatures, it is mainly due to the release of HCl in the polymer caused by heating, and the structure also changes resulting in color change. Based on the above mechanism, the colorimetric sensing ability of hydrochloric acid-treated chalcone-PDA membrane for ammonia gas was used to achieve high-sensitivity visual detection of bacterial growth and food spoilage under different temperature conditions. Compared with the existing food monitoring technology, the simple synthesis of chalcone-PDA-hydrochloric acid film, sensitive color response, and the feasibility of early visual detection of food spoilage over a wide temperature range show significant practical advantages.

**Figure 7:**
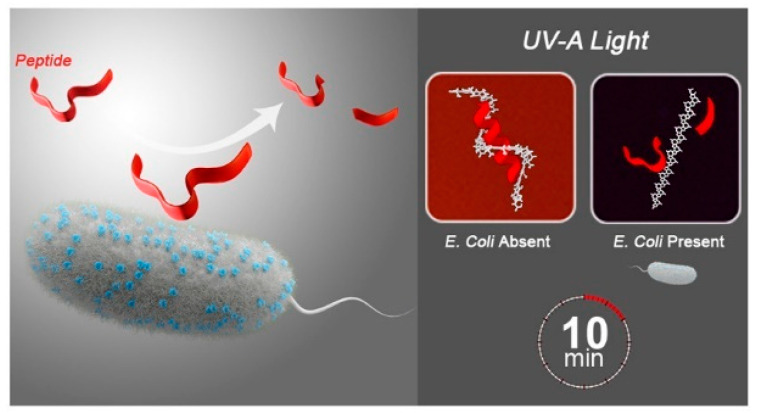
Schematic diagram of OmpT substrate-polythiophene for colorimetric detecting *E. coli*. Reprinted with permission from ref. [42]. Copyright 2022 American Chemical Society.

Sinsinbar et al. [42] reported a color change-based OmpT substrate-polythiophene assay for the detection of *E. coli* in contaminated water samples (Figure 7). This assay uses the cleavage ability of the protease (OmpT) at the double base site of the peptide to bind the substrate peptide of OmpT to polythiophene whose optical properties change with the cleavage of the peptide. *E. coli* at levels of 1–10 and 10^5^ CFU/mL were detected within 5.5 and 1 h, respectively. By optimizing the carboxylic acid side chain length of carboxylate functional polythiophene and the peptide substrate of OmpT, the detection time for *E. coli* in highly contaminated samples was reduced from the already short 1 h to only 10 min, and the colorimetric sensing ability for visual recognition under visible or UV-A light was enhanced.

## 3. Conjugated Polymers for Therapeutic Applications

### 3.1. Anti-Microorganism

#### 3.1.1. Photodynamic Therapy (PDT)

**Figure 8:**
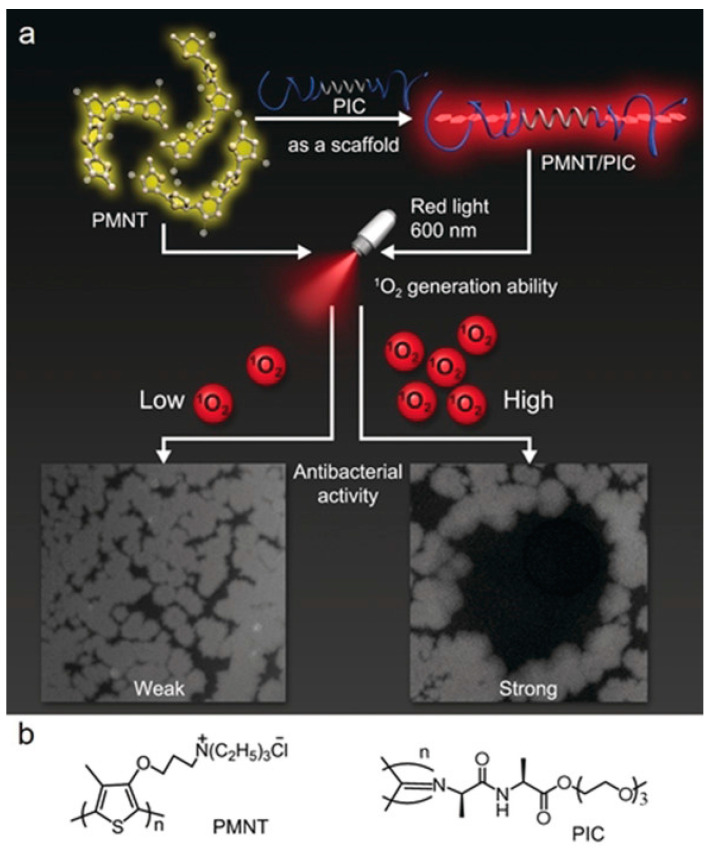
(**a**) The Illustrated assembly of PMNT/PIC for photodynamic antibacterial therapy. (**b**) The structures of PMNT and PIC. Reprinted with permission from ref. [43]. Copyright 2020 Wiley-VCH Verlag GmbH & Co. KGaA, Weinheim.

Yuan et al. [43] explored a hybrid biomimetic hydrogel based on spiral polyisocyanate and conformation-sensitive conjugated polythiophene (Figure 8). The hybrid gel uses helical triethylene glycol functionalized polyisocyanate (PIC) as a scaffold to capture and stretch the conformation-sensitive PMNT chains so that the PMNT is arranged into a highly ordered planar conformation. Thus, new absorption bands and redshifts are generated in absorption and emission spectra, respectively. PMNT/PIC hybrid hydrogels exhibit higher ROS production in random conformation than single PMNT and thus have more efficient photodynamic antibacterial activity. PMNT/PIC hybrid hydrogels retain a few of the mechanical properties of natural PIC strain hardening, which is very close to the mechanical properties of biogels such as collagen and fibrin. In addition, the hybrid hydrogels have good biocompatibility, so this PMNT/PIC hybrid material is expected to be used as wound dressings in the treatment of inner infections.

**Figure 9:**
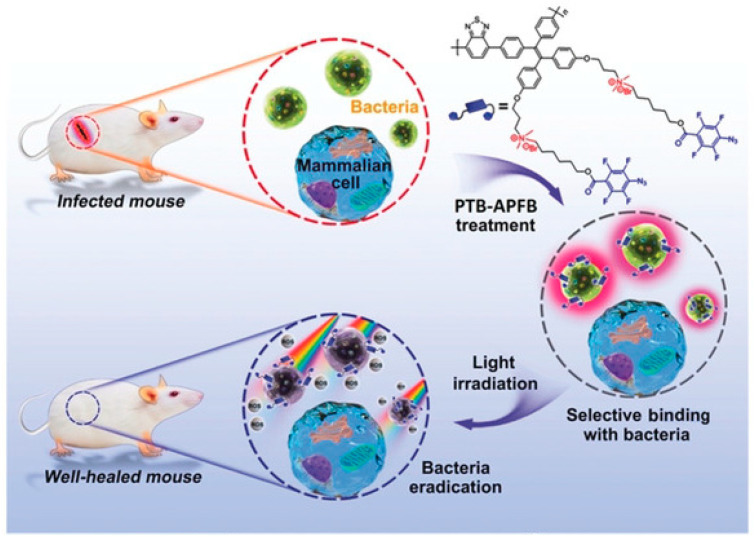
Schematic illustration of PTB-APFB for selective antibacterial application. Reprinted with permission from ref. [44]. Copyright 2020 John Wiley and Sons.

Zhou et al. [44] prepared a unique conjugated polymer (PTB-APFB) containing tetrapheny ethylene unit with aggregate induced emission (AIE) (Figure 9). Due to the donor-π-acceptor (D-π-A) structure, the polymer has a higher ROS generation capacity than the commercial photosensitizer chlorin e6. In addition, the selective binding of PTB-APFB to pathogenic microbes rather than mammalian cells demonstrated its biocompatibility. In vitro and in vivo experiments further showed that the polymer could efficiently inhibit bacterial reproduction and photodynamic killing under light irradiation, and its recovery rate for bacterial infection is faster than cephalosporin, and could effectively enhance the healing of infected wounds. Based on this, this polymer is expected to be an antibacterial drug for preclinical research and clinical application in the practical application of treating bacterial infections.

**Figure 10:**
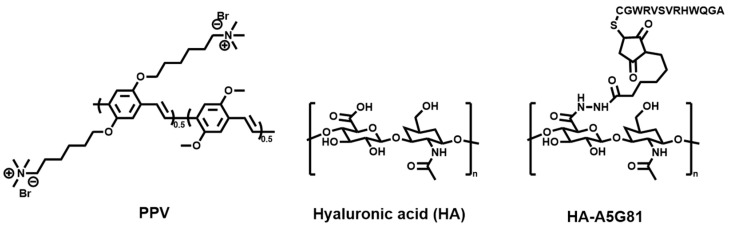
The structure of PPV, HA, and HA-A5G81 for accelerating wound repair and combating bacterial infection [45].

Zhao et al. [45] developed a 3D printable artificial hydrogel skin patch Gel/Alg/HA-A5G81/PPV comprised of natural biological macromolecules, including gelatin (Gel), alginate (Alg), and hyaluronic acid (HA), and a photoactive cationic conjugated polymer (PPV) (Figure 10). The antibacterial dressing has negligible cytotoxicity, good visible light absorption ability, strong electrostatic adsorption, and excellent adhesion ability so the artificial skin patch has good advantages of PDT against *S. aureus* infection in vitro and in vivo. In comparison with previously reported hydrogel-based dressings, the skin patch prepared in this work has comprehensive antibacterial ability, tissue regeneration ability, and rich microstructure, and is more suitable for skin equivalent in the event of skin trauma, contributing to the development of regenerative medicine.

#### 3.1.2. Photothermal Therapy (PTT)

**Figure 11:**
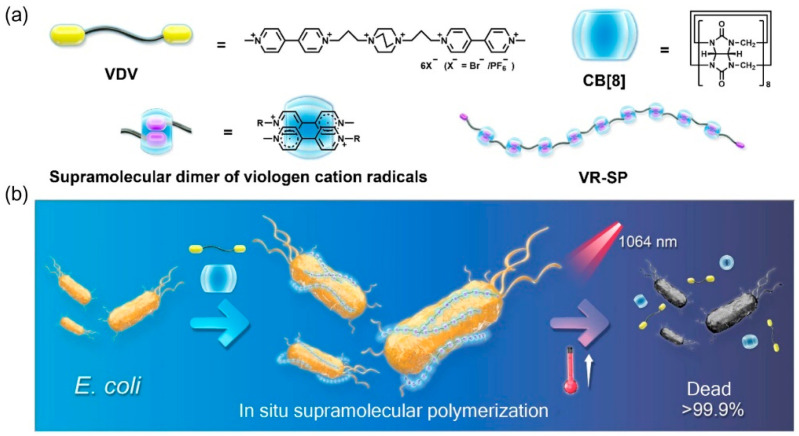
Illustration of the (**a**) supramolecular polymerization of VDV and CB [8] and (**b**) PTT based on in situ supramolecular polymerization driven by *E. coli*. This figure has been published in *CCS Chemistry* (2021); [Supramolecular Polymerization Powered by *Escherichia coli*: Fabricating a Near-Infrared Photothermal Antibacterial Agent in Situ] is available online at [https://doi.org/10.31635/ccschem.021.202101490]. Copyright 2021 Chinese Chemical Society.

Z. Yin et al. [46] reported a supramolecular polymerization driven by *E. coli* reduction to prepare a near-infrared (NIR) in situ photothermal antimicrobial agent (Figure 11). The antimicrobial agent contains two bifunctional monomers (VDV) of purpurin fragments. When the bifunctional monomer is cultured with cucurbituril (CB [8]), *E. coli* can reduce the purpurin fragments to purpurin cationic free radicals. A supramolecular polymer (VR-SP) was prepared on the surface of *E. coli*, which integrated the supramolecular dimer of violet cation radical into the main chain. The NIR photothermal conversion property of the strong free radical supramolecular dimer gives VR-SP photothermal antibacterial ability and further improves the photothermal antibacterial performance through the local enrichment of the supramolecular polymer and enhanced adsorption on the bacterial surface. In addition, only specific bacteria, such as *E. coli*, have the reducing capability to promote supramolecular polymerization, while many others, such as *B. subtilis*, *P. aeruginosa*, and *S. aureus*, do not possess this capacity. Therefore, under the NIR radiation of 1064 nm, the inhibitory effect of the supramolecular polymer on *E. coli* was significant (>99.9%), with high specificity. This biodynamic in-situ supramolecular polymerization strategy has great potential for manufacturing intelligent biomedical supramolecular materials with adaptive and programmable properties.

**Figure 12:**
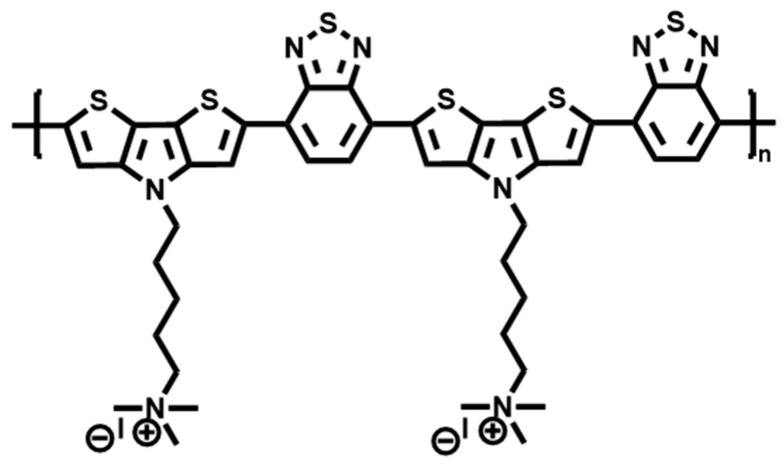
The structure of cationic conjugated polymer PDTPBT [47].

Zhang et al. [47] designed and synthesized a cationic conjugated polymer (PDTPBT) based on a D-A structure, which was composed of dithieno[3,2-b:2′,3′-d]pyrrole (DTP) as electron donor and benzothiadiazole as electron acceptor, respectively, which was used for photothermal antimicrobial treatment under NIR light. In PDTPBT polymer (Figure 12), QAS groups are modified on the side chain to improve the water solubility and effectively promote the electrostatic binding with bacteria. PDTPBT has strong NIR absorption performance, good light stability, and high photothermal conversion efficiency (PCE) of up to 71.1%. Therefore, the novel cationic conjugated polymer PDTPBT has good photothermal properties and high antibacterial ability, which gives a viable strategy for the development of organic photothermal agents and the biomedical applications of NIR light sterilization.

**Figure 13:**
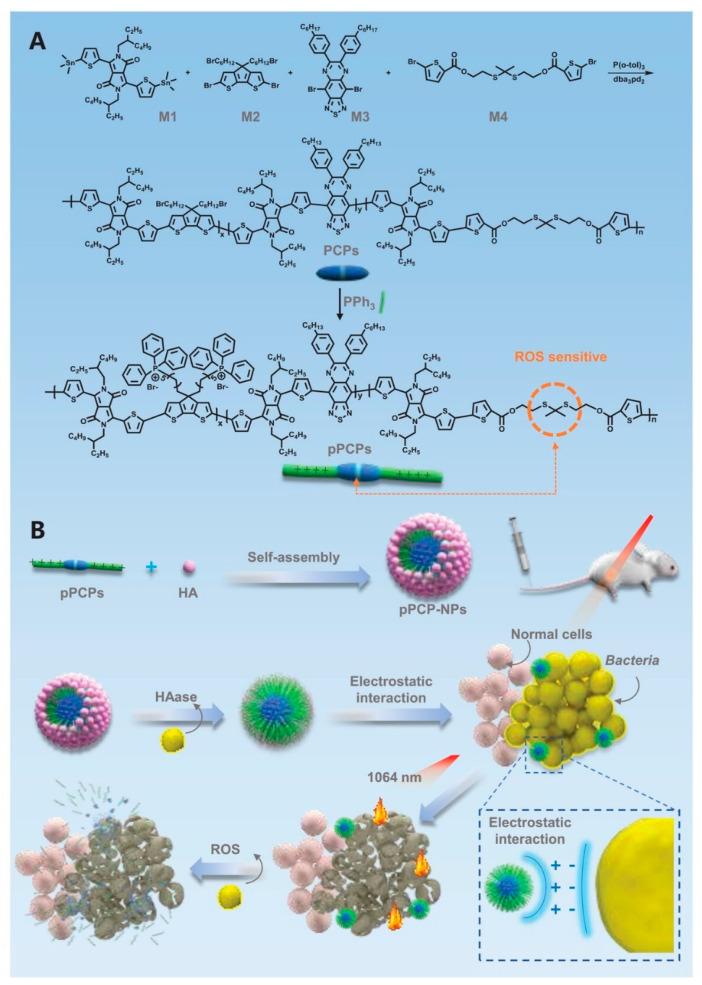
Schematic diagram of (**A**) the synthesis of pPCPs and (**B**) pPCP-NPs for photothermal antibacterial treatment. Reprinted with permission from ref. [48]. Copyright 2022 The Authors.

PTT based on CPs is a promising antimicrobial strategy, but it may still have some disadvantages such as difficult degradation. Zhou et al. [48] designed a biodegradable pseudo-conjugated polymer (pCPs) comprised of a photothermal molecular skeleton and ROS-sensitive thioketone bond (Figure 13). Triphenylphosphine (PPh_3_) was connected to PCP to form phosphonium-based PCPs (pPCPs), followed by electrostatically combining with hyaluronic acid to obtain pPCP nanoparticles (pPCP-NPs). pPCP-NPs with phosphonium cation can specifically anchor bacterial cell membranes through electrostatic interaction and have a certain destructive effect. In addition, under 1064 nm laser irradiation, pPCP-NPs produces NIR-II region (1000–1700 nm) photothermal antibacterial effects, thus killing pathogens continuously. Compared with photothermal agents with excitation wavelength in near-infrared region I (NIR-I: 650–950 nm) and antibacterial agents without quaternary ammonium salt modification, the coordination of NIR-II photothermal agent and the cation of phosphonium salt have better antibacterial effect, the inhibition rate against both of *S. aureus* and *E. coli* in vitro is close to 100%, and the repair effect of infection in vivo is also very satisfying. pPCP-NPs can be degraded in vivo by a lot of ROS present at the infection site, which enhances its biosafety. Further metabolomic analysis showed that pPCP-NPs with the aid of NIR light induced bacterial DNA damage and inhibited bacterial carbon/nitrogen utilization and amino acid/nucleotide synthesis. Therefore, the biodegradable pPCP-NPs with photothermal effect and quaternary phosphonium salt anchored bacteriostatic effect propose an alternative way to replace antibiotics.

#### 3.1.3. Synergistic Antibacterial Therapy

**Figure 14:**
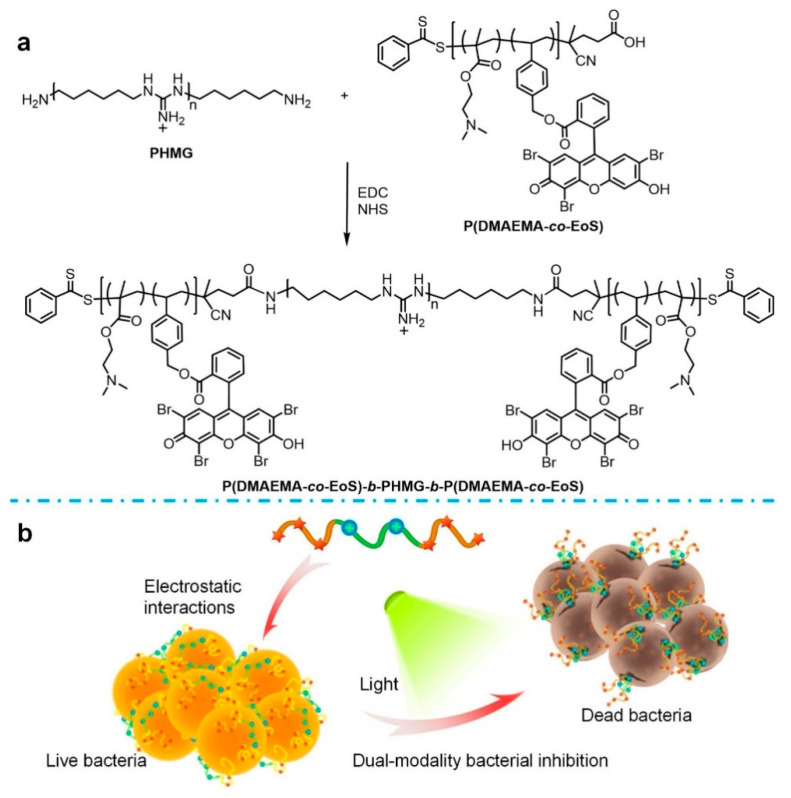
The schematic diagram of PEoS-PHMG (**a**) synthesis for (**b**) dual-mode inhibition of bacteria through synergistic PDT. Reprinted with permission from ref. [49]. Copyright 2020 Published by Elsevier B.V. on behalf of the Chinese Chemical Society and Institute of Materia Medica, Chinese Academy of Medical Sciences.

The effect of photodynamic or photothermal treatment alone is affected by low oxygen concentration or high temperature, respectively, and the effect of synergistic treatment through multiple methods is much stronger than that of using one method alone [73,74,75]. Xiao et al. [49] developed a broad-spectrum polymer antimicrobial agent PEoS-PHMG, which achieves dual-mode antibacterial effects through a combination of photodynamic inactivation and physical damage (Figure 14). A typical commercial cationic antimicrobial agent (PHMG) was selected to be linked in the polymer, which quickly adsorbed to the negatively charged cell surface through electrostatic action and damaged the cell membrane by increasing the permeability of the cell plasma membrane to the site to form local pores for intracellular components leaking and eventually led to physical damage and bacterial death [76,77]. This damage is beneficial to avoid causing bacterial drug resistance [78,79]. In addition, PHMG also has excellent photodynamic bactericidal ability, with a dual antibacterial mechanism to effectively kill a broad spectrum of bacteria. PHMG is then combined with Eosin-Y (EoS) to obtain polymer P(DMAEMA-*co*-EoS), followed by copolymerization with 2-(dimethylamino) ethyl methacrylate to obtain a new antibacterial polymer agent PEoS-PHMG. Compared with single PHMG, conjugated functionalized PHMG shows enhanced biocompatibility and good bacterial inhibition. Therefore, functionalized PHMG is a prospective strategy for the exploration of novel polymer photodynamic antimicrobials.

**Figure 15:**
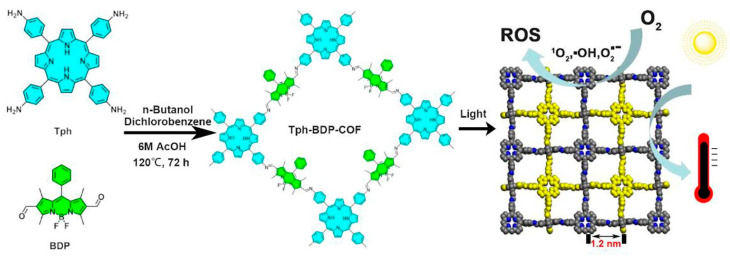
Schematic diagram of Tph-BDP-COF for ROS generation and photothermal conversion under light irradiation. Reprinted with permission from ref. [50]. Copyright 2022 Elsevier B.V.

Zhao et al. [50] synthesized a covalent organic framework (Tph-BDP-COF) containing porphyrin (Tph) and borodipyrrole (BDP) derivatives based on the combined antibacterial strategy of PDT and PTT (Figure 15). When ROS is present, protective heat shock proteins at high temperatures are inhibited by biochemical reactions [80,81] in other words, the production of ROS reduces the cell heat tolerance and thus achieves high-temperature sterilization. To adjust the dissipation of excitation energy, excellent synergistic antibacterial phototherapy is achieved. Tph-type photosensitizers have good light collection ability, produce ROS through electron and energy transfer, and generate heat from molecular vibration under light. However, the strong absorption of Tph is mainly at 400–450 nm, and effective photothermal production requires strong absorption in the NIR region. The absorption band of BDP itself is in the visible light range, but the photophysical properties will change greatly after modification. Therefore, combining the modified BDP unit with the Tph derivative to form a high-porosity COF structure that facilitates oxygen infiltration and ROS release enabling excellent Vis-NIR absorption, which in turn generates ROS and heat. Compared with the two mono-molecules, the large redshift absorption band in the NIR region of the composite proves that the NIR absorption is obtained due to the strong charge transfer (CT) interaction of COF. In addition, the composite conjugated polymer has a narrow optical band gap (1.33 eV) and rapid energy and electron transfer, which greatly promotes the ISC process and improves the photothermal conversion efficiency, and the integration of multiple antibacterial mechanisms can shorten the antibacterial time and improve the antibacterial efficiency. By photodynamic and photothermal treatment, Tph-BDP-COF showed highly efficient light-induced synergistic antibacterial properties (97%). These results not only provide inspiration for the design of new synergistic therapy photosensitizers under the guidance of Jablonski, but also provide hope for further exploration of excited state dynamic control of phototherapeutic materials.

**Figure 16:**
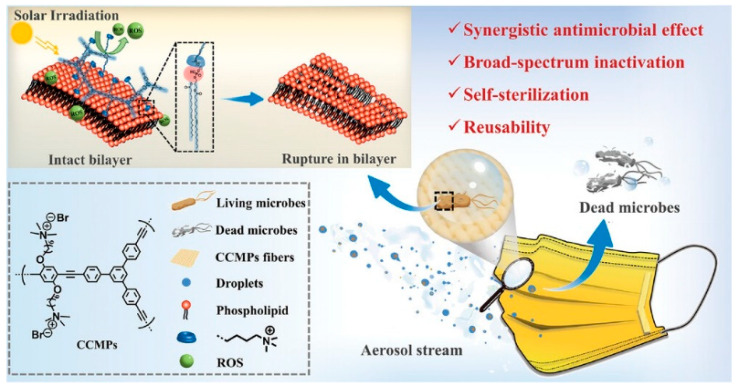
Illustrated microbial inactivation of CCMPs. Reprinted with permission from ref. [51]. Copyright 2023 Wiley-VCH GmbH.

In the context of the COVID-19 pandemic, face masks are considered important personal protective equipment to prevent the spread of the viruses. However, pathogens can survive on the fabric of commercial masks for several days, which increases the risk of direct/indirect transmission. Wang et al. [51] developed a novel cationic conjugated microporous polymer (CCMPs) coating containing an extended π-conjugated backbone and a large number of QAS groups with dual-mode antimicrobial activity containing photodynamic sterilization by ROS and contact sterilization by QAS groups (Figure 16). The CCMPs coating can quickly and effectively eradicate 99% of representative microorganisms such as *E. coli* and *S. aureus* under sunlight and also guarantees good antibacterial effects in the dark. Moreover, CCMPs coating displays superior durability, reusability, and antimicrobial stability in wet environments. Due to their excellent figurability, CCMPs can be synthesized in situ and coated onto the fibers by simply spraying. Therefore, the strategy offers new thinking for exploring reusable and self-disinfecting antibacterial textiles, especially for use in masks for daily and medical protection to combat pathogens and viruses.

### 3.2. Antitumor Application

#### 3.2.1. Photodynamic Antitumor Therapy

**Figure 17:**
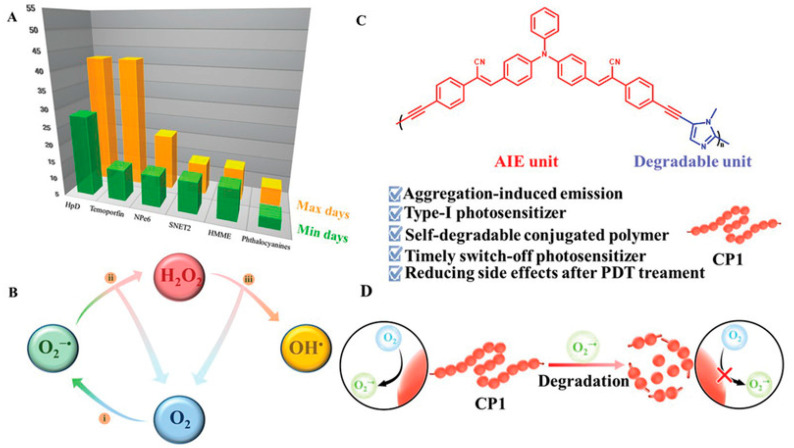
(**A**) The treatment time statistics of commercial photosensitizers. (**B**) The illustrated cascade reaction of O_2_^−•^ in PDT. (**C**) The structure of CP1 and (**D**) the schematic diagram of O_2_^−•^ generated by CP1 before and after degradation. Reprinted with permission from ref. [52]. Copyright 2021 The Authors.

Huang et al. [52] proposed a photosensitizer (PS) for type I PDT based on a self-degrading CP, which consists of triphenylamine and imidazole units (CP1) (Figure 17). As mentioned in the introduction, the type I photodynamic process mainly produces ROS of free radicals, which is less dependent on oxygen than the type II PDT process which produces ROS of ^1^O_2_, thus alleviating the problem of hypoxic microenvironment in the treatment area [82]. Owing to the conjugated backbone and unique AIE properties, the resulting polymer could efficiently produce superoxide free radicals (O_2_^−•^) via a type I way under light, making it desirable for the treatment of hypoxic tumors. Furthermore, the production of O_2_^−•^ from CP1 could further cause the polymers to self-degrade and form non-toxic micromolecules. This not only aids in solving the problem of potential phototoxicity of residual PS, but also improves the metabolism of CPs and decreases the possible biological toxicity of drug accumulation. This self-degrading type I PS can shut down ROS production timely after photodynamic treatment, giving a new insight for balancing photodynamic effects and postoperative safety.

**Figure 18:**
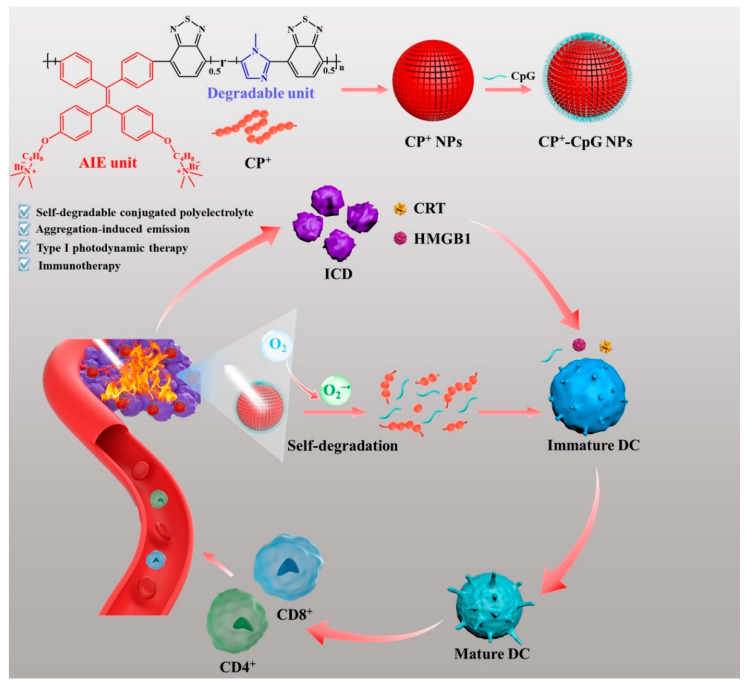
The illustrated preparation of CP^+^-CpG NPs and the synergy of PDT and immunotherapy. Reprinted with permission from ref. [53]. Copyright 2022 Published by Elsevier B.V.

In the same year, Huang et al. [53] designed a nanoparticle assembled by self-degrading conjugated polyelectrolyte (CP^+^) followed by loading immune adjuvant (CpG) to obtain CP^+^-CpG NPs for the coordination of PDT and immunotherapy (Figure 18). During the PDT process, ROS can cause endoplamic reticulum oxidative stress and lead to immunogenic cell death (ICD). Nevertheless, most PDT-caused ICDs could not generate sufficient anti-tumor immune responses to inhibit tumor metastasis. CP^+^-CpG NPs could efficiently produce O_2_^−•^ under light irradiation via the type I PDT way which is ideal for combating tumors in hypoxic conditions. Meanwhile, CP^+^-CpG NPs could be degraded by self-produced O_2_^−•^, thereby releasing CpG which could further enhance PDT-caused ICD, activate an immune response, and bring about the entire body’s immune anti-tumor reactions. The new strategy enhances the effect of PDT and induces immunotherapy, which holds great promise in future cancer treatment.

**Figure 19:**
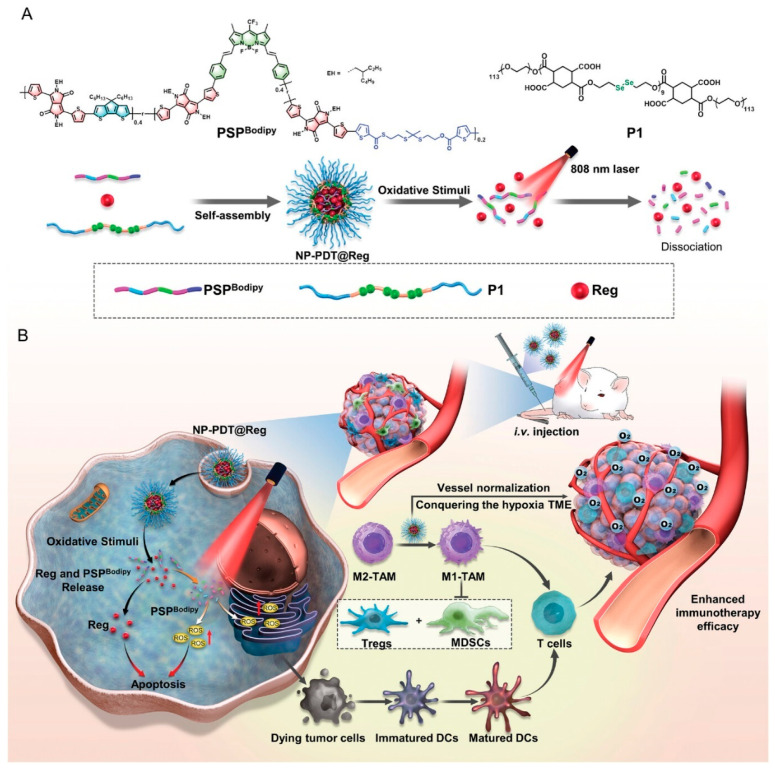
The illustrated (**A**) preparation and (**B**) application of NP-PDT@Reg for photodynamic immunotherapy. Reprinted with permission from ref. [54]. Copyright 2023 Wiley-VCH GmbH.

The effect of PDT is restricted by hypoxic tumors, which are closely associated with tumor vascular abnormalities that promote immune evasion. Thus, tumor vascular standardization is considered a prospective method to surmount tumor hypoxia and thus improve tumor treatment. Wan et al. [54] reported a NIR-II-absorbing biodegradable pseudo-conjugated polymer-based photodynamic material (PSP) to deliver a vascular normalizer (Reg) resulting in nanoparticles (NP-PDT@Reg) (Figure 19). Reg can be effectively released from NP-PDT@Reg under NIR light to improve hypoxia in tumors through vascular standardization, allowing more nanoparticles and oxygen to enter the tumor. In addition, NP-PDT@Reg with the aid of NIR light could further lead to the production of more ROS to eliminate tumor cells and induce ICD to stimulate immune responses for anti-tumor. Furthermore, Reg could reprogram tumor-associated macrophages (TAM) from the pro-tumor M2 phenotype to the tumor-killing M1 phenotype, hence leading to a changeover of the tumor microenvironment that is immunosuppressive. The investigation gives insight into the exploration of promising nanomaterials to conquer the shortcomings of photodynamic immunotherapy.

#### 3.2.2. Photothermal Antitumor Therapy

**Figure 20:**
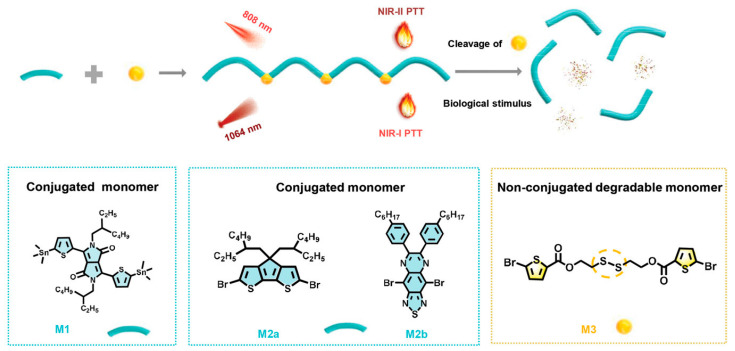
Synthesis of biodegradable PTA and its application in PTT anti-tumor schematic diagram. Reprinted with permission from ref. [55]. Copyright 2021 Wiley-VCH GmbH.

Yu et al. [55] synthesized a new series of biodegradable polymers containing a flexible structure with disulfide bonds that can be cleaved by glutathione (GSH) which is the most abundant in animal cells. These biodegradable polymers are prepared through the Stille polymerization of conjugated monomers containing tin (M1) and monomers containing bromine (M2a, M2b, and M3). In such systems, the conjugated part gives the polymers photothermal conversion capability, while the biodegradable non-conjugated part gives the system biosafety. This design strategy provides inspiration for the development of effective photothermal agents (Figure 20).

**Figure 21:**
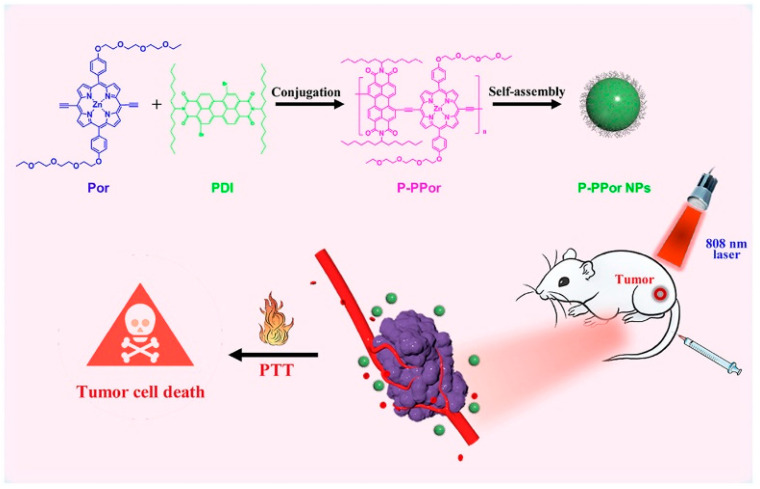
Synthesis of P-PPor NPs and schematic diagram of photothermal anti-tumor application. Reprinted with permission from ref. [56]. Copyright 2021 Published by Elsevier Ltd.

Li et al. [56] reported a porphyrin polymer (P-PPor) with a D-A structure (Figure 21). P-PPor exhibits strong absorption in the NIR-I region and emits significant fluorescence in the NIR-II region with a quantum yield of 2.19%. Because of the hydrophilic PEG chains and hydrophobic alkyls in the conjugated backbone, amphiphilic P-PPor can form well water-dispersed nanoparticles (P-PPor NPs) via self-assembly with improved absorption in the NIR region. In addition, P-PPor NPs displayed quenching fluorescence due to the aggregation-caused quenching (ACQ) effect, and the photothermal effect was produced and remarkably enhanced. Under 808 nm laser irradiation, P-PPor NPs have a high PCE of 66%. The excellent photothermal efficacy of P-PPor NPs was further verified by the 4T1 tumor model. At the same time, in vivo NIR-II fluorescence imaging showed a high distribution of P-PPor NPs at the tumor site. It is indicated that P-PPor NPs can efficiently kill mouse tumor cells under NIR light without obvious side effects. Therefore, P-PPor NPs can be utilized as a promising drug for the photothermal treatment of tumors with high safety and effectiveness.

**Figure 22:**
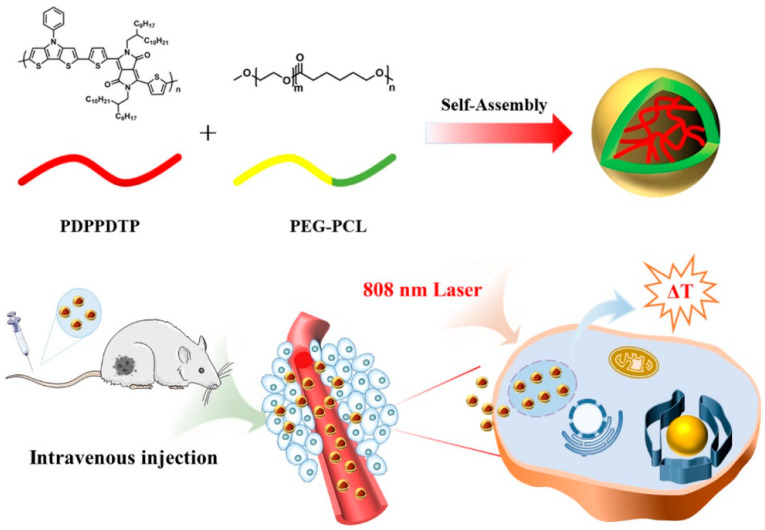
Illustration of nano-micelles for PTT. Reprinted with permission from ref. [57]. Copyright 2022 American Chemical Society.

Zhang et al. [57] prepared a novel D-A conjugated polymer PDPPDTP based on a pyrrole derivative as the electron-donating unit and a pyrrole-1,4-dione derivative as the electron-accepting unit (Figure 22). The obtained polymer has strong absorption in the NIR-I range and exhibits effective photothermal properties under 808 nm laser irradiation. The biodegradable amphiphilic polymer polyethylene glycol polycaprolactone (PEG-PCL) was utilized to wrap the novel PDPPDTP into nano-micelles. The nano-micelles have excellent biocompatibility and low cytotoxicity, and can efficiently restrain the proliferation of 4T1 cells under 808 nm light irradiation. In addition, in vivo photothermal treatment indicated that nano-micelles loaded PDPPDTP displayed significant tumor growth inhibition in isogenic mouse tumor models, demonstrating its potential as a novel therapeutic drug.

#### 3.2.3. Synergistic Antitumor Therapy

**Figure 23:**
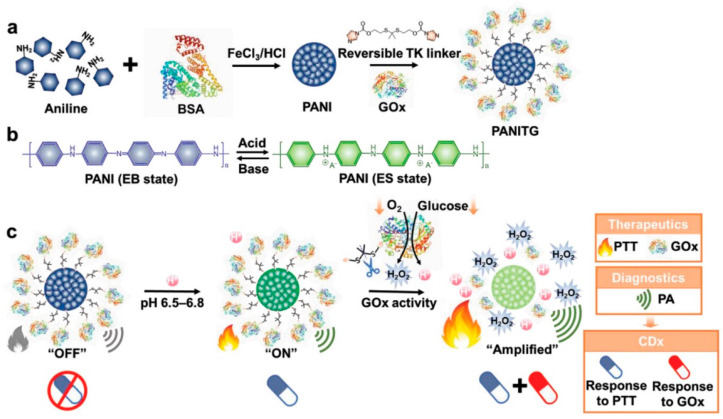
Scheme of PANITG nanoplatform (**a**) preparation and (**b**) reaction mechanism for (**c**) CDx and multistage enhanced synergistic therapy. Reprinted with permission from ref. [58]. Copyright 2022 Wiley-VCH GmbH.

Companion diagnostics (CDx) can provide vital information for precision medicine, but currently, CDx with imaging function is rare and limited to in vitro trials, which cannot accurately assess disease progression and treatment response in real-time. To solve this problem, Wu et al. [58] reported a glucose oxidase (GOx) modified conjugated polymer nanoparticle (PANITG) for acid-activated photoacoustic (PA) imaging CDx and multistage enhanced synergistic photothermal/starvation treatment of tumors (Figure 23). PANITG is composed of polyaniline (PANI), GOx, and thioketal (TK) linkers that can be cut by H_2_O_2_. PANI synthesized by traditional methods has poor water solubility and poor dedoping resistance in the tumor microenvironment. Therefore, this work adopts the first oxidation of polyaniline. It is then combined with FeCl_3_ biomineralized bovine serum albumin complex to form PANI NPs, and GOx is modified on its surface by TK linker. The protonated emerald salt (ES) of PANI NPs has strong absorption in the NIR light region under an acidic environment due to its pH-dependent electrochromic properties. In neutral or alkaline environments, its deprotonated emerald (EB) has low NIR light absorption and can be used as a PA imaging “switch” to distinguish between diseased and normal tissue. PANI NPs can also produce a photothermal effect, and local hyperthermia can lead to tumor ablation. Activated PTT can avoid non-specific overheating of surrounding normal tissues during phototherapy. However, the pH changes in the tumor microenvironment were relatively weak compared with normal tissues (H_2_O_2_ content increased ≈ 0.1 × 10^−6^ M, pH decreased to pH = 6.5–6.8), so the photothermal treatment effect of PANI NPs alone was limited. When PANITG combined with GOx catalyzes glucose oxidation, it can not only compete with cancer cells for glucose demand, but also induce cancer starvation therapy. At the same time, the oxidation reaction product glutamic acid enhances the acidity of the tumor microenvironment, and the oxidation product H_2_O_2_ can cut the TK linker and release GOx. The enhanced acidic environment enhances the catalytic activity of free GOx, further enhancing the acidity of the tumor environment and the consumption of glucose, and the reduction of pH enhances the effect of PA, PTT, and starvation therapy. Compared with free GOx, the activity of GOx on PANITG is inhibited, so it does not cause toxicity to normal cells. Only in the tumor microenvironment does H_2_O_2_ release TK linker after cutting to restore its catalytic glucose oxidation activity, and further realizes starvation therapy to kill tumor cells. Therefore, PANITG has good tumor treatment selectivity and biocompatibility. In vivo anti-tumor experiments showed that PANITG achieved tumor ablation in mice for 14 days under NIR light, but had no effect on normal tissue. Overall, in vivo PAI and photothermal therapeutics are stimulated by the acidic microenvironment in tumors and self-enhanced by the reaction of GOx with glucose. Meanwhile, the photothermal effect will improve GOx activity, and this multi-stage enhanced therapeutic effect effectively promotes cancer management. In addition, the signal brightness of PANITG in vivo PAI shows the pH of the tumor, which correlates with the efficacy of PTT, and the catalytic activity of GOx at various stages, thus revealing pH-activated/enhanced PTT and GOx-mediated starvation therapy progression, enabling reactivable real-time CDx.

**Figure 24:**
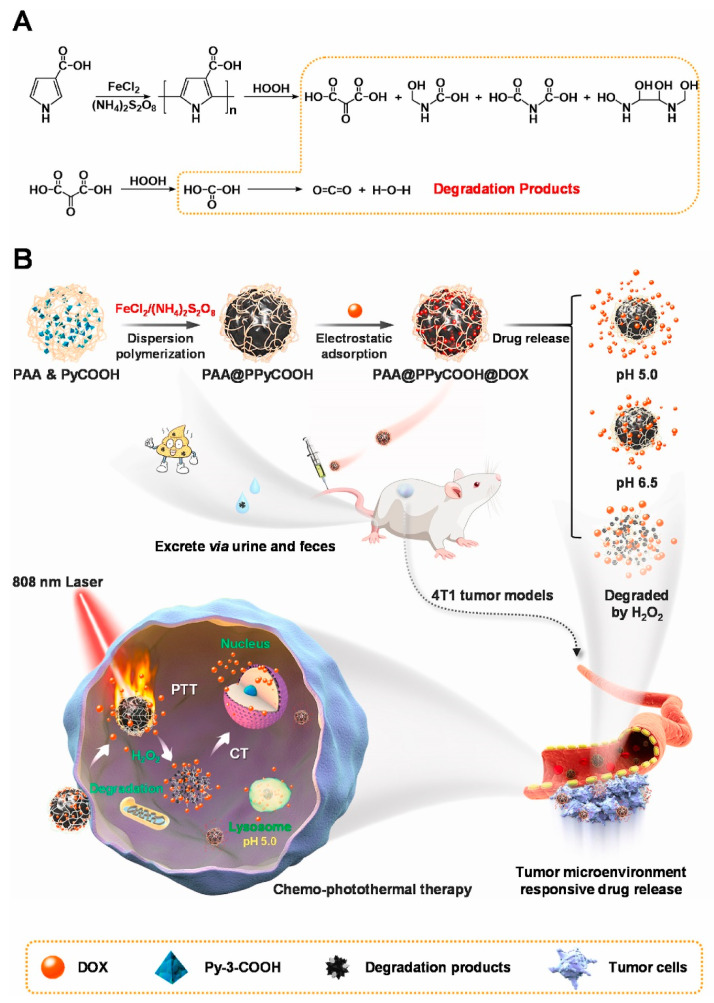
Illustrated (**A**) synthesis of PAA@PPyCOOH@DOX for (**B**) chemo-PTT. Reprinted with permission from ref. [59]. Copyright 2021 Elsevier Ltd.

Conductive polymers have strong NIR absorption and have received extensive attention to developing smart nanoplatform for anti-tumor therapy, especially photothermal chemotherapy. Nevertheless, due to the non-degradability of conductive polymers, their long-term biosafety in vivo is unknown, which hinders their clinical application. Liu et al. [59] synthesized a polypyrrole derivative that is degradable by H_2_O_2_-triggering, and the corresponding nanoparticles are prepared with the stabilization of polyacrylic acid (PAA) to obtain PAA@PPyCOOH which has good dispersibility, biocompatibility and high PCE (56%) (Figure 24). Subsequently, nanoparticles (PAA@PPyCOOH@DOX) were formed with the addition of doxorubicin (DOX) for precise synergistic chemo-PTT. PAA@PPyCOOH@DOX releases DOX in the acidic and H_2_O_2_ overexpressed microenvironment in dual response, resulting in an excellent chemical photothermal treatment effect. PAA@PPyCOOH could be degraded through the ring-opening reaction of the pyrrolo-3-carboxyl unit in the presence of H_2_O_2_. When nanoparticles are selectively decomposed by excess H_2_O_2_ in tumors, their decomposition products can be eliminated through urine and feces. The anti-tumor results of chemotherapy-PTT in vivo showed that tumor growth was significantly restrained, and no obvious side effects were found, which has great clinical application potential.

**Figure 25:**
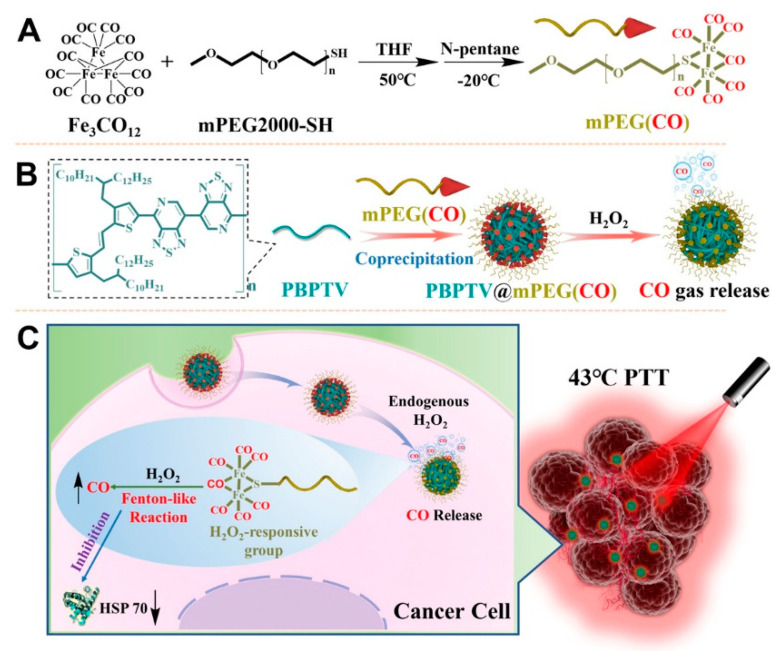
Illustrated preparation of (**A**) mPEG(CO) and (**B**) PBPTV@mPEG(CO) and (**C**) low-temperature therapy of nanobomb. Reprinted with permission from ref. [60]. Copyright 2022 Wiley-VCH GmbH.

Low-temperature PTT overcomes the disadvantages of traditional PTT such as poor heat tolerance, and the expression of heat shock proteins (HSPs) can significantly affect the treatment effect of PTT. Therefore, preventing the repair of heat shock proteins and reducing the damage of nearby normal cells is the key to improving the efficiency of low-temperature PTT. Ma et al. [60] developed a self-assembled nanobomb based on NIR-II polymer PBPTV and carbon monoxide (CO) carrier polymer mPEG(CO) (Figure 25). The smart nano-bomb could explode in the tumor microenvironment with overexpression of H_2_O_2_ and release CO into tumor cells, thus efficiently inhibiting the expression of HSPs and improving the anti-tumor effects of low-temperature PTT. This method provides an ideal strategy for the development of tumor hypothermia therapy and exhibits great potential in clinical practice.

### 3.3. Integration of Diagnosis and Treatment Based on CPs

#### 3.3.1. Detection and Treatment of Pathogenic Microorganisms

**Figure 26:**
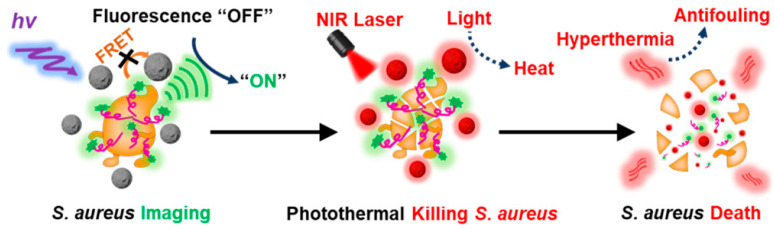
Schematic diagram of nano-probe PDANSs-FAM-Apt used for the imaging-guided photothermal killing of *S. aureus*. Reprinted with permission from ref. [61]. Copyright 2020 American Chemical Society.

The effective integration of precise tracking and on-demand sterilization for source control of pathogenic bacteria is of great significance to be explored. Ye et al. [61] reported a stimulus-responsive nanoprobe PDANSs-FAM-Apt for the detection of *S. aureus* at the single-cell level and direct photothermal killing of *S. aureus* and its biofilm under NIR light (Figure 26). Polydopamine nanospheres (PDANSs), which are used for FRET receptors due to their wide absorption bands, are connected to the *S. aureus* aptamer modified with the FRET donor 6-carboxyfluorescein (FAM-Apt) via π-π stacking interactions to obtain probe PDANSs-FAM-Apt, and the fluorescence of the donor is quenched. However, in the presence of *S. aureus* that specifically absorbs the aptamer, the π-π accumulation is reduced, and the FRET between PDANSs and FAM-Apt is broken, followed by the fluorescence recovery of FAM-Apt to realize the sensitive detection of *S. aureus*. Subsequently, the nanosphere size is adjusted to maximize the FRET efficiency according to the dependence of FRET efficiency on the size of PDANSs, thus maximizing the sensitivity of the probe. The results showed that the probe displayed significant fluorescence enhancement (261 times) for *S. aureus*, and the detection limit was 1.0 CFU/mL, enabling the detection of pathogens at the single-cell level and ultra-low background fluorescence imaging. In addition, due to the high photothermal conversion efficiency of PDANSs, the probe exhibits high photothermal bactericidal performance under NIR light irradiation and has a strong killing ability against *S. aureus* and its biofilm under the guidance of imaging. This work emphasizes the versatility of the combination of stimulus-response fluorescence imaging and NIR light-activated photothermal antibacterial activity, which can be used for pathogen monitoring and source control, opening up a practical anti-fouling pathway for the elimination of bacteria and destruction of bacterial biofilms.

**Figure 27:**
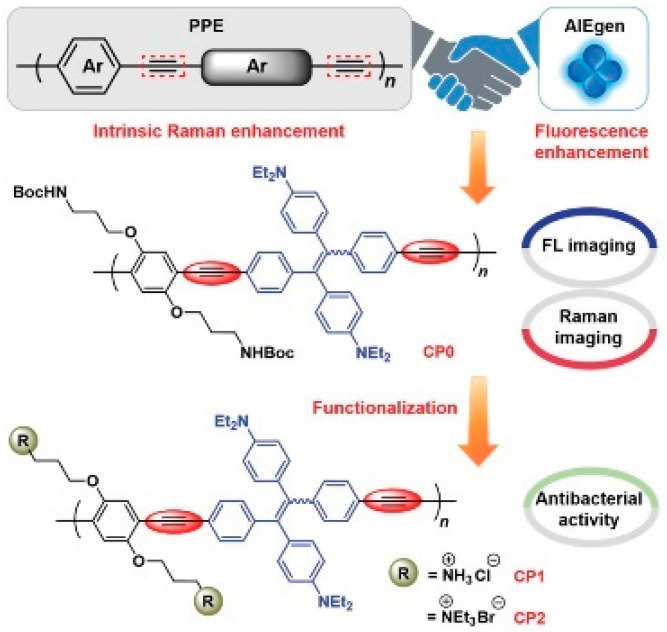
Illustrated design of AIE-active PPEs. Reprinted with permission from ref. [62]. Copyright 2021 Wiley-VCH GmbH.

Su et al. [62] synthesized a series of functional conjugated polymer poly(phenyleneethynylene) (PPE) derivatives containing diethylamino-substituted tetraphenylethene units, with typical AIE properties (Figure 27). PPE nanoparticles based on hydrophobic structure have good cellular uptake capacity and can clearly and sensitively observe the presence of bacteria through fluorescence and Raman signals, respectively. After functionalization, the obtained cationic PPE can preferentially image bacteria rather than mammalian cells and has excellent photodynamic inactivation ability toward G^+^ bacteria and drug-resistant bacteria. This report gives a viable strategy for the exploration of functional CPs with multimodal imaging capabilities and photodynamic antimicrobial capabilities.

**Figure 28:**
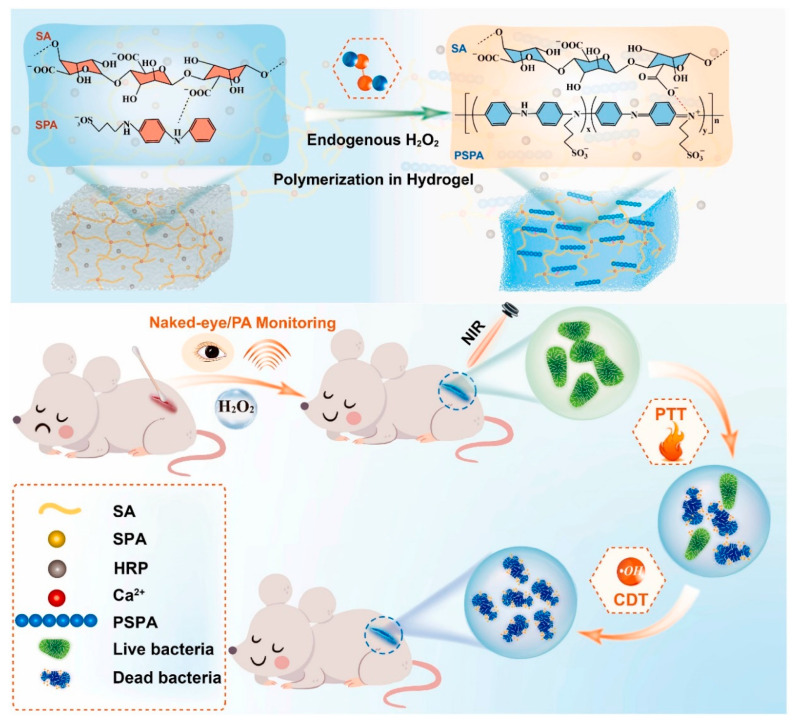
Schematic diagram of in-situ polymerization of H_2_O_2_-activated SPA in hydrogel for real-time monitoring and treatment of wound infection. Reprinted with permission from ref. [63]. Copyright 2022 Elsevier Ltd.

It is important to develop an effective integrated treatment system that simultaneously possesses the functions of real-time monitoring of wound status and the timely treatment of wound infections. Wan et al. [63] used the biological microenvironment for the first time to successfully in situ synthesize conjugated polymers in artificial hydrogels, realizing real-time monitoring and bacterial inhibition of wound infection (Figure 28). The conjugated polymer hydrogel consists of an easily polymerized aniline dimer derivative (SPA), which is skillfully polymerized in situ in calcium alginate (CA) hydrogel to form PSPA. The PSPA that preloads horseradish peroxidase (HRP) could catalyze H_2_O_2_ overexpressed in infected wounds to produce •OH, which leads to oxidative killing bacteria. The NIR absorption of PSPA enables the real-time monitoring of H_2_O_2_ with the naked eye and photoacoustic signals and has a photothermal inhibition effect on bacteria mediated by NIR light. In addition, combined with the sustained chemokinetic activity of •OH, in vivo treatment demonstrated a wound healing rate of 99.03% on day 11. Thus, this study provides inspiration for manipulating in situ biosynthesis of functional CPs in artificial hydrogels to solve multiple problems in wound treatment.

#### 3.3.2. Detection and Treatment of Tumor

**Figure 29:**
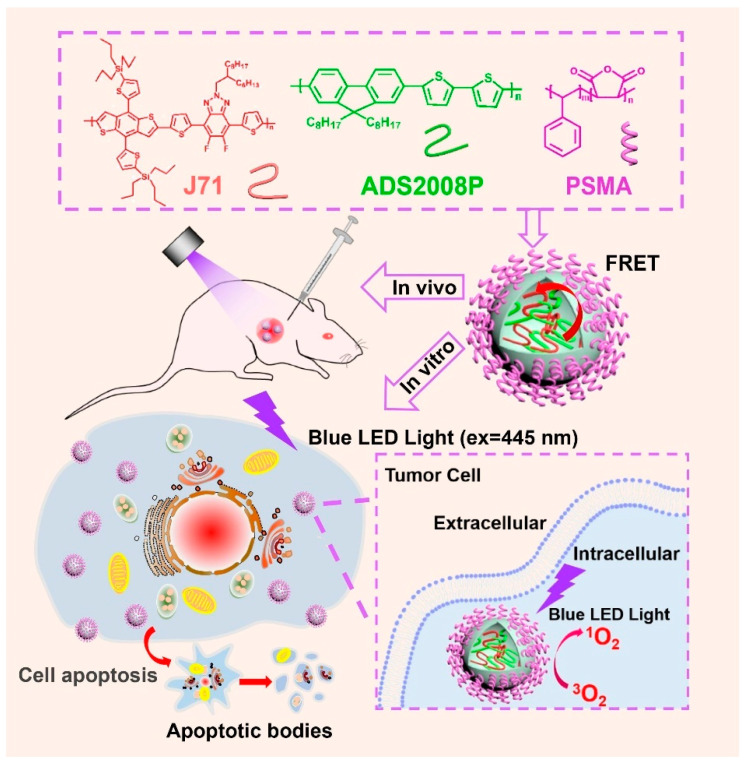
Illustrated preparation of ADS2008P/J71 NPs for imaging-guided PDT. Reprinted with permission from ref. [64]. Copyright 2022 Elsevier B.V.

In the process of tumor imaging-guided PDT, how to achieve both strong fluorescence and high ROS production efficiency under light excitation is the key to realizing the integration of diagnosis and treatment. Therefore, it is a challenge to construct a single photosensitizer integrating the advantages of high quantum yield, efficient singlet oxygen production, stable red-light emission, excellent photostability, and low dark toxicity. Zhao et al. [64] designed an efficient D-A system with superior imaging and photodynamic therapeutic capabilities by combining an active polymer (J71) with a hydrophobic conjugated polymer (ADS2008P) to prepare the multifunctional nanoparticles (ADS2008P/J71 NPs) (Figure 29). Through the FRET from ADS2008P to J71, the singlet oxygen production yield of the nanoparticles is increased to 0.704 accompanied by a high fluorescence efficiency. In addition, as a new photosensitizer, J71 is a two-dimensional conjugated polymer, which has good photostability compared with traditional porphyrin photosensitizers. The results of in vivo and in vitro experiments show that the system has excellent fluorescence imaging ability and significant anti-tumor capacity, indicating that the system has great application prospects in imaging-guided PDT.

**Figure 30:**
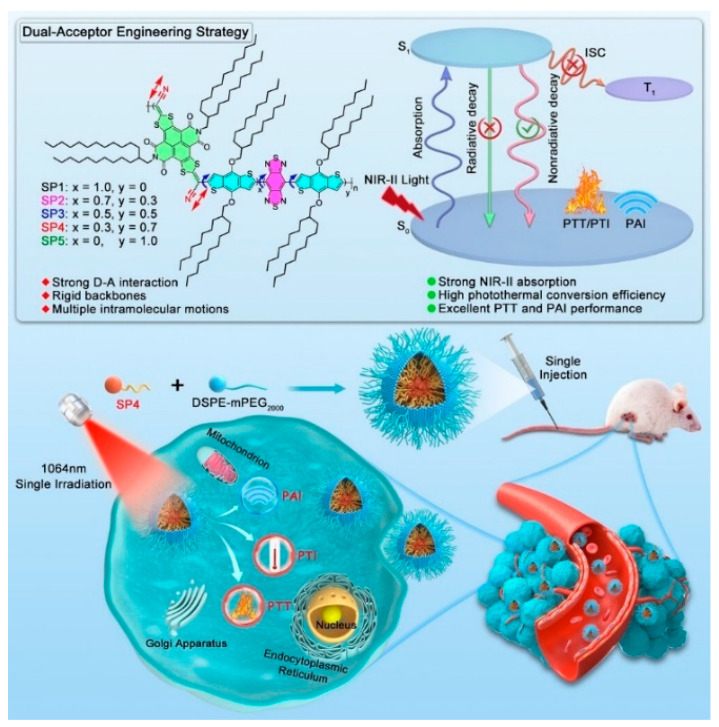
Illustrated design of SPs and the PAI-guided PTT. Reprinted with permission from ref. [65]. Copyright 2023 Wiley-VCH GmbH.

Li et al. [65] synthesized a series of D-A-type semiconductor polymers (SPs), using a precise double-acceptor strategy to fully utilize intramolecular motion to induce photothermal effects by adjusting the molar ratio of two acceptors with different electron affinity and π electron delocalization degree (Figure 30). The best polymer SP4 has good NIR-II absorption, a high extinction coefficient, and excellent photothermal conversion properties. As a result, SP4 NPs have unprecedented performance in 1064 nm laser-stimulated PAI-guided PTT to accurately diagnose and completely eliminate the tumor. This work integrates ultra-long excitation wavelength, high extinction coefficient, and excellent photothermal conversion capability into a single photothermal agent, which enriches the database of NIR-II absorbing SPs and offers further exploration strategy of multifunctional PTA design for the photothermal treatment of cancer.

## 4. Conclusions and Prospects

Due to the excellent photoelectric properties, good biocompatibility, and easy degradation of CPs with π-conjugated system, functional CPs can meet the fluorescence/colorimetric detection of pathogens and fluorescence/photoacoustic imaging of tumors. In addition, under the activation of the appropriate wavelength of light, CPs can be excited to produce ROS and heat that can oxidize and kill cells through different dissipation pathways. These two processes correspond to PDT and PTT, respectively. With the emergence of drug-resistant bacteria, the antibiotic era will be replaced, and at the same time, antimicrobials and treatments that can avoid resistance will become urgent. The CPs with ingenious design strategies summarized and discussed above can meet the antibacterial needs of phototherapy only by using PDT or PTT. For example, adding the QAS group can improve the water solubility of CPs and their ability to combine with the cell membrane by carrying positive charges, and it also can make CPs biodegradable by introducing chemical bonds such as thioketone bond and disulfide bond which are sensitive to a specific environment. Therefore, CPs are one of the most potential organic biochemical materials to replace antibiotics in biomedicine. However, the antibacterial/anti-tumor activity of CPs will be limited by the hypoxia environment and heat shock protein when PDT or PTT is used alone. For this reason, many studies now combine different treatment methods to achieve synergistic antibacterial or anti-tumor effects and give full play to the therapeutic effect of each functional unit of the material to meet the needs of targeted and efficient multi-purpose.

Furthermore, with the continuous development of the application of CP materials, existing research results have put forward a comprehensive strategy to coordinate the sensing/imaging of pathogens and tumors with the treatment process. This design idea can put the theoretical assumption of instant detection and timely treatment into practice and is expected to dynamically guide the treatment process through imaging and sensing methods, which plays a strong role in promoting the application of CPs in the biomedical field, making CPs be a multi-purpose material with further research prospects.

Although much progress has been made, there are still some limitations and potential challenges for CPs-based detection and treatment. For example, the relationship between the structure/size and functionality of CPs needs further exploration to clarify the correlation between them. Secondly, the strategy of designing CPs that can control the molecular weight within the absorption and metabolism range of the human body or be degradable, and at the same time have the ability of efficient diagnosis and treatment is still worthy of attention and research. In addition, there are many subjects that need to be studied and experimented before it is put into clinical use. Among them, biological safety, such as the exploration of the transportation, distribution, and metabolism of most water-soluble CPs in the body, the in-depth understanding of the metabolic mechanism, and the long-term safety and toxicity in the body need further evaluation. In a word, it is still a challenge to transform biomedical applications based on CPs (such as detection imaging, drug delivery detection, and phototherapy) into clinical applications, and it takes a long way to go and a certain time to realize.

## Figures and Tables

**Table 1:** Overview of the application of CPs in the biomedical field.

Application	Method		Polymer Category	Highlight	Reference
Pathogen detection	Fluorimetry		Conjugated oligomer electrolyte	Specific detection of G^+^ in mixed samples to realize Gram typing	[37]
			Polyfluorene derivative	Rapid and sensitive identification of bacteria and fungi in mixed pathogenic samples through the different binding force	[38]
			Polythiophene (PT)	The modified QAS group increases water solubility and gives a positive charge	[39]
	Colorimetry		Poly(3,4-propylenedioxythiophen-alt-3,4-ethylenedioxythiophene) copolymer (PPE)	Discrimination of G^−^ and G^+^ by detecting the color change produced by the reduction of bacterial metabolites.	[40]
			Polydiacetylene (PDA)	Treat with hydrochloric acid then react with ammonia gas metabolized by bacteria, and change color with different polymerization degrees by adjusting monomer arrangement.	[41]
			Protease (OmpT) substrate-PT	Through the color change of polythiophene with the cleavage of peptide, the unit level *E. coli* was detected colorimetrically within 10 min	[42]
Antibacterial therapy	PDT		PT + functionalized polyisocyanate (PIC)	The conformational sensitive CP is arranged in a highly ordered conformation to produce more ROS	[43]
			Poly(tetrapheny ethylene)	Have AIE characteristics and higher ROS production ability	[44]
			Poly(phenylenevinylene) (PPV)	Have a positive charge and excellent PDT effect on *S. aureus*	[45]
	PTT		Purpurin + cucurbituril (CB [8])	Supramolecular polymerization driven by *E. coli* reduction in situ, the inhibitory effect on *E. coli* was >99.9%	[46]
			Copolymerization of dithieno[3,2-b:2′,3′-d]pyrrole (DTP) and benzothiadiazole	Have D-A structure and QAS groups, increase water solubility, and impart positive charge, PCE = 71.1%	[47]
			Biodegradable pseudo-CP	The ROS in the infected site breaks the thioketone bond sensitive to ROS to achieve biodegradability	[48]
	Synergistic therapy	PDT + physical damage	Cationic conjugated polymer	Double bacteriostatic mechanism, broad-spectrum antibacterial	[49]
		PDT + PTT	Porphyrin (Tph)-borodipyrrole (BDP)-COF	The production of ROS reduces the cell heat tolerance and thus achieves high-temperature sterilization	[50]
		PDT + QAS groups	Cationic conjugated microporous polymer	Quickly and effectively eradicate 99% of representative microorganisms under sunlight	[51]
Antitumor therapy	PDT		Copolymerization of triphenylamine and imidazole	type I PDT, the production of O_2_^−^• further degrades the polymer into nontoxic micromolecules, shutting down ROS production timely after PDT	[52]
			Conjugated polyelectrolyte	Type I PDT, PDT-caused ICD	[53]
			Multi-monomer co-polymerization with ROS-sensitive thio-ketone bond	Carrying vascular normalization agent can make more oxygen enter the tumor area, improve tumor hypoxia microenvironment, improve PDT effect, and induce ICD	[54]
	PTT		Biodegradable CP	Can be cleaved by glutathione (GSH) in mammalian cells	[55]
			Porphyrin polymer (P-Ppor)	Targetability, NIR-II PDT	[56]
			Copolymerization of 2,6-di(trimethyltin)-N-dithieno[3,2-b:20,30-d]pyrrole and 3,6-bis(5-bromothiophen-2-yl)-2,5-dihexadecylpyrrole	Can efficiently restrain the proliferation of 4T1 cells under 808 nm light irradiation	[57]
	Synergistic therapy	PTT + starvation treatment	glucose oxidase (Gox)-polyaniline (PANI)	Gox competes with cancer cells for glucose to form starvation therapy, and oxidation products make the tumor microenvironment acidic, which leads to enhanced PTT effect, PTT also enhances Gox activity	[58]
		PTT + chemoth-erapy	Polypyrrole derivative	H_2_O_2_-triggered degradation, the double-response release of DOX by acidity, and H_2_O_2_ excess	[59]
		PTT + Gas therapy	Conjugated polymer + carbon monoxide (CO) carrier polyethyleneglycol (mPEG)	Excessive H_2_O_2_ in the tumor microenvironment leads to the release of CO to inhibit HSPs expression and realize low-temperature PTT	[60]
Detection and treatment of pathogen	Fluorescence + PTT		PDA	NIR PDT, fluorescence was quenched before binding with *S. aureus*, and FRET was broken after binding to restore green fluorescence to realize the detection at the single cell level	[61]
	Fluorescence and Raman signals + PDT		Poly(phenyleneethynylene) (PPE)	AIE characteristics enhance fluorescence, excellent PDT effect on G^+^	[62]
	naked eye/PAI + PTT + CDT		Polymerization of aniline dimer derivative	Preload HRP catalyzes H_2_O_2_ to produce •OH, wound healing rate in vivo on day 11 to 99.03%	[63]
Detection and treatment of tumor	Fluorescence + PDT		Combine poly[(9,9-dioctylfluorenyl-2,7-diyl)-alt-co-(bithiophene)] and photosensitizer (J71)	Strong fluorescence and high ROS generation efficiency are realized under the excitation of visible light	[64]
	PAI + PTT		Copolymerization of benzo[1,2-c : 4,5-c′]bis([1,2,5]thiadiazole (BBTD) and 2-(1,3-dithiol-2-ylidene)acetonitriles (NDI-DTYA2)	NIR-II absorption, photothermal imaging diagnosis (PTI), precise treatment	[65]

## Data Availability

Data sharing not applicable.
